# Supplementary material for: A global cancer data integrator reveals principles of synthetic lethality, sex disparity and immunotherapy
Source: Genome Med. 2021 Oct 18;13:167. doi: 10.1186/s13073-021-00987-8 (PMC8524992; doi:10.1186/s13073-021-00987-8)
Supplement: Supplementary file 1 — Additional file 1: Figures S1-3. Figure S1. An Object-oriented schema diagram showing core structure of CanDI software v0.2-alpha. Gene, CellLine, Oraganelle, Cancer, and CellLine classes all inherit from the Entity Class. Figure S2. A scatter plot comparing gene essentiality (Average CERES scores) of NSCLC cell lines between KRAS mutant and KRAS wild type Cell lines with EGFR mutant cell lines removed from consideration. Figure S3. A scatter plot comparing gene essentiality (Average CERES scores) of NSCLC cell lines between EGFR mutant and EGFR wild type Cell lines with KRAS mutant cell lines removed from consideration. [file 13073_2021_987_MOESM1_ESM.docx]

**ADDITIONAL FILE 1**

 **Figure S1.** An Object-oriented schema diagram showing core structure of CanDI software v0.2-alpha. Gene, CellLine, Oraganelle, Cancer, and CellLine classes all inherit from the Entity Class. The Entity class contains methods for data filtration and attributes of data transforming objects. The Data class contains load and unload methods and attributes of either data file paths for pandas dataframes. Data attributes are accessed and transformed via Entity attributes and methods.

**Figure S2.** A scatter plot comparing gene essentiality (Average CERES scores) of NSCLC cell lines between KRAS mutant and KRAS wild type Cell lines with EGFR mutant cell lines removed from consideration.

**Figure S3.** A scatter plot comparing gene essentiality (Average CERES scores) of NSCLC cell lines between EGFR mutant and EGFR wild type Cell lines with KRAS mutant cell lines removed from consideration.
